# Editorial statement

**DOI:** 10.1007/s10611-020-09898-y

**Published:** 2020-04-28

**Authors:** Mary Dodge

**Affiliations:** School of Public Affairs, University of Colorado Denver, 1380 Lawrence St., Ste 500, Denver, CO 80217 USA

The success of *Crime, Law and Social Change* (CL&SC) depends on a large work group including Springer’s team, reviewers, and managing editors. Additionally, we acknowledge and appreciate the significant contributions made by our editorial board members. Because individually thanking each member who has served the journal would be a Promethean task, this special introduction attempts to acknowledge the past and present dedication of our editorial board. Our recent efforts to invite new scholars to serve on the board have resulted in commentary that I would like to share.

Many board members have agreed to continue in their current role. Michael Johnston, for example, who brings years of historical experience to our collective work commented:

I have been a board member since godknowswhen, have enjoyed the work, and look forward to continuing to contribute. In fact, I have a deeper history with CL&SC: back in the mid-1980s I co-founded the journal *Corruption and Reform,* published by Martinus Nijhoff, with two close UK colleagues. We operated on a virtual shoestring for seven years, producing a journal that reflected a continuing rise in quality, and that was out ahead of the curve in terms of interest in corruption issues. "Ahead of the curve" isn't always a good place to be, however; in early 1993 (!) Nijhoff, claiming there was insufficient interest in corruption research, told us they were folding our journal into *CL&SC,* then edited by Alan Block. Eventually my good friend Nikos Passas invited me to join the board.

Also, Peter Yeager will remain on the board, despite his gracious gesture to step aside: “I am happy to continue in this role. But I am also happy to relinquish it to another qualified person… whom membership on the board would constitute valuable experience and exposure as well as a helpful achievement in a rising career arc.” My belief is that we can accommodate current members and add new scholars as suggested by Peter. John Braithwaite, now Emeritus, has agreed to continue participating and responded in a lovely personal message that needs no further explanation for those who know the brilliance of this celebrated scholar.

Professor Thomas Naylor, Department of Economics at McGill University, offered insight into the journal’s history. He mentioned a number of issues--past, present, and future--that serve as reminders of CL&SC’s high quality and trailblazing approach. And he indicated his preference for publishing in our journal. Dr. Naylor also contributed numerous invaluable articles while Alan Block and Nikos Passas were editors. Many of Dr. Naylor’s comments noted the innovative nature of our articles and remind us of a timeless guiding principle:

Looking back, I was just about the only person who was nominally an economist working with your journal or even in criminology. [T]hat was a good thing since most of what economists have to say today is best ignored since it can be harmful if anyone really takes it seriously. Most of my generation of troublemakers have moved on. I hope CL&SC can continue to stand outside the mainstream of social science—since the mainstream has always looked to me more like a cesspool.

Dr. Naylor’s comments remind us that, at times, academic muckrakers are essential to discovering new “truths.” Our hope is that we can apply his message and strive to be innovative. Dr. Naylor will be sorely missed as a member of our editorial board.

Michael Lynch, who was appointed to the board in 1994 shared an important message from William J. Chambliss, who founded the original journal (*Contemporary Crises*). In 1977, Bill (a friend and colleague wholeheartedly missed by our entire community) offered these enduring words:

[C]hanging and challenging ideas bespeak the fact that social science is currently experiencing an intellectual revolution of gigantic proportions. Theoretical traditions are being challenged and new paradigms are being explored. The period is both the most exciting and the most frustrating for perhaps the past fifty years. It is exciting, for the prospects are bright for breaking out of the ideological and pseudo-scientific strait-jackets that have hampered rather than pushed forward the quest for knowledge (p. 1).

We would be remiss to ignore Bill’s call for further “innovation in the emerging intellectual traditions” that promote critical criminology. Remembering Bill’s contributions and his sociological imagination inspires us to remain the leader in publishing international articles that challenge current paradigms and offer cutting edge research.

We thank and honor all our current and departing editorial board members who have wisely served the journal for many years. Also, we welcome our new board members: Rita Faria, Universidade do Porto; Rajeev Gundur, Flinders University; Olga Petintseva, Ghent University and Melissa Rorie, Univeristy of Nevada—Las Vegas. Megan J. Parker, University of Colorado Denver, will be serving as our new book review editor and Sari Weichbrodt will continue as managing editor.

I find myself writing this introduction during self-imposed isolation, but I remain hopeful that as we weather COVID-19 our world becomes a better place of continued caring and understanding. Best wishes to our staff, editorial board members, contributors, and readers who make us a premier journal. Stay safe and I hope to see many of you at the European Society of Criminology in Romania.

Mary Dodge, Co-Editor in Chief.

